# Covalent targeting of PSMD14 by Eupalinolide B induces oncoprotein degradation and apoptosis in acute promyelocytic leukemia cells

**DOI:** 10.1039/d5cb00197h

**Published:** 2026-02-04

**Authors:** Zheng Chu, Liting Xu, Honglin Chen, Tianyun Fan, Xueqian Hu, Yin Kwan Wong, Qiaoli Shi, Junzhe Zhang, Chengchao Xu, Jigang Wang, Huan Tang

**Affiliations:** a State Key Laboratory for Quality Ensurance and Sustainable Use of Dao-di Herbs, Artemisinin Research Center, and Institute of Chinese Materia Medica, China Academy of Chinese Medical Sciences Beijing 100700 China ccxu@icmm.ac.cn jgwang@icmm.ac.cn htang@icmm.ac.cn; b Department of Pulmonary and Critical Care Medicine, Shenzhen Institute of Respiratory Diseases, Guangdong Provincial Clinical Research Center for Geriatrics, Shenzhen Clinical Research Center for Geriatrics, Shenzhen People's Hospital, The Second Clinical Medical College, Jinan University Shenzhen 518020 Guangdong China; c Department of Biological Sciences, National University of Singapore Singapore 117543 Singapore; d Department of Oncology, Ningbo Municipal Hospital of Traditional Chinese Medicine Affiliated to Zhejiang Chinese Medical University Ningbo 315010 China

## Abstract

Treatment of acute promyelocytic leukemia (APL) remains challenged by toxicities associated with current regimens, highlighting the need for novel and safer therapeutic agents. Here, we identify Eupalinolide B (EB), a natural sesquiterpene lactone isolated from *Eupatorium lindleyanum DC.*, as a potent anti-leukemic compound targeting the human APL-derived HL-60 cell line. Through integrated chemoproteomic profiling and functional validation, we demonstrate that EB covalently binds and inhibits 26S proteasome non-ATPase regulatory subunit 14 (PSMD14), a deubiquitinase enzyme (DUB) within the 19S proteasome regulatory particle. This inhibition disrupts PSMD14-mediated stabilization of key oncoproteins RAC-alpha serine/threonine-protein kinase 1 (AKT1) and cyclin-dependent kinase 4 (CDK4), promoting their proteasomal degradation. As a result, EB induces G2/M cell cycle arrest and apoptosis in leukemia cells. Both genetic knockdown and pharmacological inhibition of PSMD14 recapitulate EB's effects, confirming its essential role in leukemia cell survival and proliferation. Collectively, these findings uncover a previously unrecognized PSMD14–AKT1/CDK4 regulatory axis in leukemia and position EB as a promising chemical probe and lead compound for the development of targeted covalent inhibitors against oncogenic DUBs.

## Abstract 2

Eupalinolide B covalently targets PSMD14, inhibiting its deubiquitinase activity. This leads to degradation of oncoproteins AKT1 and CDK4, resulting in cell cycle arrest and apoptosis in leukemia cells.

## Graphical abstract

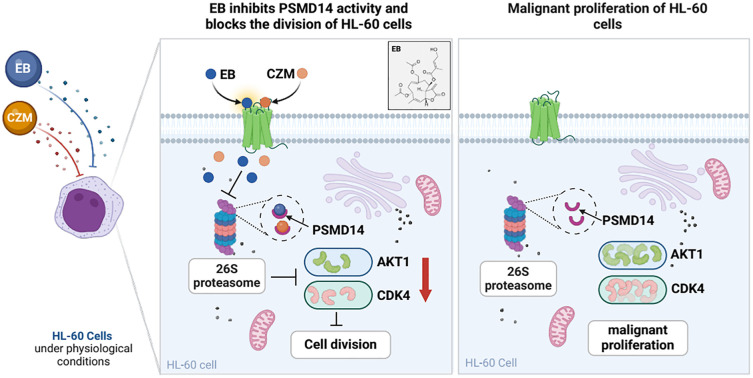

## Introduction

Acute promyelocytic leukemia (APL), a unique form of acute myeloid leukemia (AML), arises from the malignant transformation of promyelocytes, resulting in uncontrolled proliferation, impaired differentiation, and suppression of normal hematopoiesis.^1,2^ Clinically, this aggressive disease often presents with life-threatening complications, most notably a high risk of hemorrhagic diathesis and disseminated intravascular coagulation (DIC) due to a characteristic coagulopathy.^2,3^ Patients may also experience severe anemia, recurrent infections, organomegaly, and debilitating bone pain.^3^ The advent of all-*trans* retinoic acid (ATRA) in combination with arsenic trioxide (ATO) has dramatically transformed the prognosis of APL, converting it from a highly fatal condition to one of the most curable forms of leukemia.^3–5^ Nevertheless, despite this therapeutic success, several limitations remain. Treatment with ATRA/ATO is associated with significant toxicities, including ATRA syndrome, arsenic-induced neurotoxicity, cardiotoxicity, and the potential risk of secondary malignancies.^3,5,6^ Furthermore, the burden of intensive chemotherapy and the need for hematopoietic stem cell transplantation (HSCT), as required for other AML subtypes,^1,4^ highlight the urgent need for novel therapeutic agents. Such agents should ideally maintain high efficacy while minimizing toxicity, ultimately improving patient outcomes and quality of life.

Natural products, especially those derived from medicinal plants, constitute a valuable source of novel therapeutic agents, as exemplified by the landmark discoveries of artemisinin and paclitaxel.^7^ Among these, *Eupatorium lindleyanum DC.* has long been utilized in traditional Chinese medicine (TCM) for the treatment of inflammatory respiratory disorders, including bronchitis, cough, and pneumonia.^7^ Recent phytochemical studies have identified Eupalinolide B (EB), a characteristic sesquiterpene lactone, as a principal bioactive compound responsible for the plant's anti-inflammatory properties.^7,8^ Beyond its anti-inflammatory effects, EB has demonstrated a broad spectrum of pharmacological activities. Notably, it exhibits significant neuroprotective effects by covalently targeting deubiquitinating enzymes such as USP7, thereby alleviating neuroinflammation.^9^ Furthermore, EB has shown potent cytotoxic effects against multiple cancer cell lines, including solid tumors (*e.g.*, A549, BGC823, SMMC7721) and, notably, the human APL cell line HL-60.^12^ Despite these promising findings, the precise molecular mechanisms underlying EB's anticancer effects, particularly its cytotoxicity against leukemia cells and its potential as a therapeutic agent for APL, remain poorly understood. This gap in knowledge presents a major barrier to the rational development and clinical translation of EB or its derivatives for leukemia treatment.

To elucidate the molecular mechanisms underlying EB's anti-leukemic effects, we employed an integrated approach combining phenotypic screening with advanced chemoproteomic target deconvolution. Using the human APL-derived HL-60 cell line as a clinically relevant model, we first confirmed that EB robustly induces apoptosis and promotes G2/M phase cell cycle arrest. Subsequent chemoproteomic profiling, utilizing an activity-based EB probe (EB-P), identified the 26S proteasome regulatory subunit PSMD14, a JAB1/MPN/Mov34 (JAMM) family deubiquitinase enzyme (DUB), as the primary covalent intracellular target of EB. Importantly, we demonstrated that EB binding functionally inhibits the deubiquitylase activity of PSMD14. This inhibition accelerates the degradation of key oncogenic proteins, specifically RAC-alpha serine/threonine-protein kinase 1 (AKT1) and cyclin-dependent kinase 4 (CDK4), both of which are stabilized by PSMD14 and are critical for HL-60 cell proliferation. The consequent downregulation of AKT1 and CDK4 underlies the observed cell cycle arrest and apoptotic response. Overall, these findings establish EB as a novel covalent inhibitor of the DUB PSMD14 and uncover a previously unrecognized mechanism by which PSMD14 promotes leukemia cell survival through stabilization of AKT1 and CDK4. Targeted inhibition of PSMD14 by EB thus represents a promising therapeutic strategy for the treatment of APL.

## Results

### EB induces apoptosis and G2/M cell cycle arrest in HL-60 cells

**Fig. 1:**
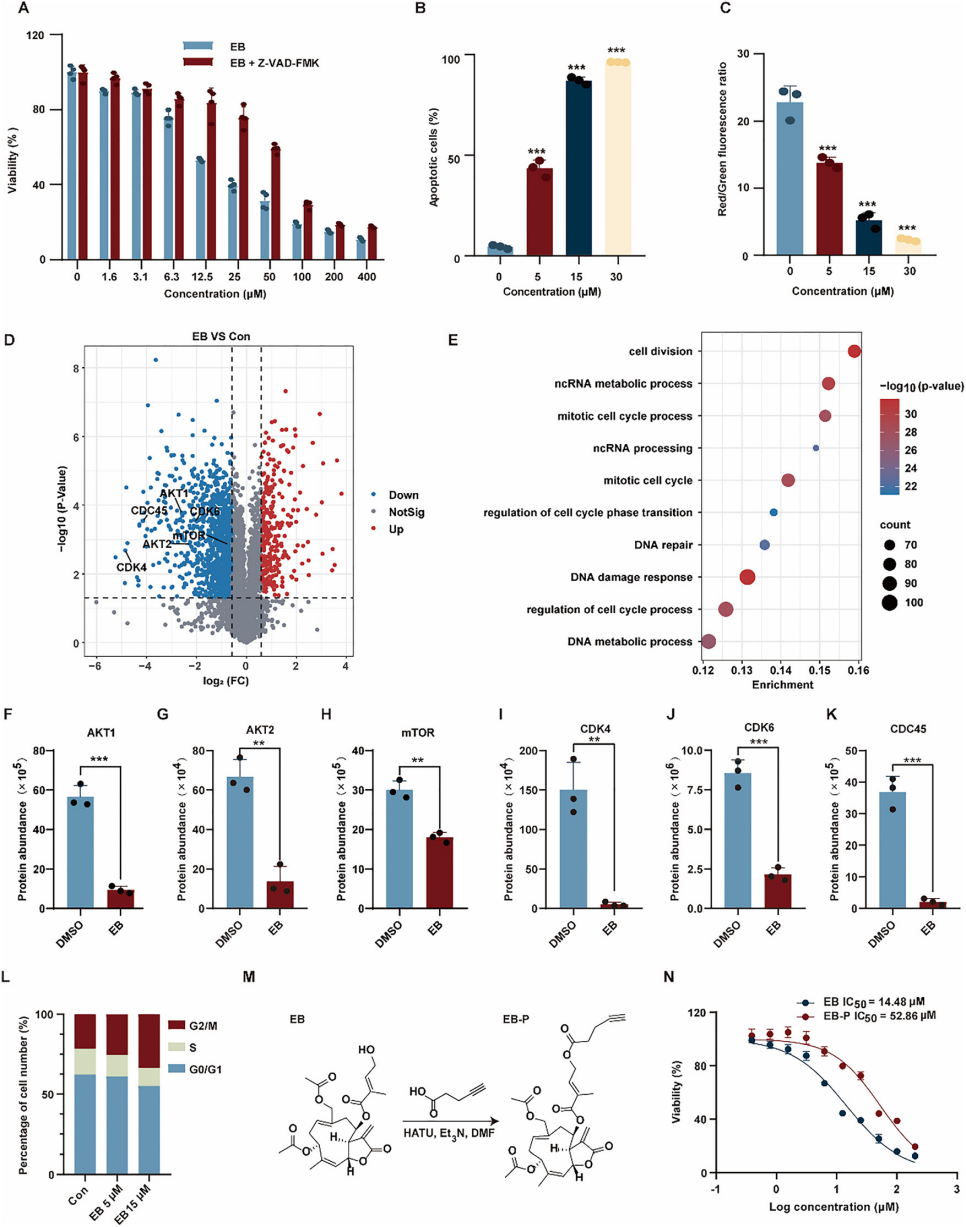
(A) Z-VAD-FMK reversed EB-induced apoptosis in HL-60 cells. (B) EB induced apoptosis in HL-60 cells. (C) EB reduced mitochondrial membrane potential in HL-60 cells. (D) Volcano plot showing EB-induced alterations in protein expression in HL-60 cells; differentially expressed proteins (DEPs) were identified using a cutoff of fold change > 1.5 and *P* < 0.05. (E) Gene Ontology (GO) enrichment analysis of proteins downregulated by EB; the top 10 pathways with the lowest *P*-values are displayed. (F–K) EB significantly downregulated proteins associated with cell cycle and division, including AKT1 (F), AKT2 (G), mTOR (H), CDK4 (I), CDK6 (J), and CDC45 (K). (L) EB significantly disrupted cell cycle progression in HL-60 cells. (M) Chemical synthesis of the EB-derived probe (EB-P). (N) Anti-proliferative activity of EB and EB-P in HL-60 cells. ** *P* < 0.01, *** *P* < 0.001.

Initial evaluation of EB's cytotoxicity in HL-60 cells revealed a dose-dependent decrease in cell viability (Fig. 1A). The partial rescue of cell viability following co-incubation with Z-VAD-FMK (a pan-caspase inhibitor) suggests that EB-induced cell death occurs primarily *via* apoptosis (Fig. 1A). This conclusion was further supported by a concentration-dependent increase in apoptotic cell populations (Fig. 1B and Fig. S1A) and a concurrent loss of mitochondrial membrane potential (Δ*Ψ*_m_) (Fig. 1C and Fig. S1B), both well-established markers of apoptosis. To explore the molecular mechanisms underlying EB's cytotoxic effects, we performed global proteomic profiling of EB-treated HL-60 cells. Volcano plot analysis, using thresholds of fold change (FC) >1.5 and *p* < 0.05, identified 1015 significantly downregulated proteins compared to control cells (Fig. 1D). Gene Ontology (GO) enrichment analysis of these downregulated proteins revealed their predominant involvement in essential cellular processes such as cell division, regulation of cell cycle process and DNA damage response (Fig. 1E). Notably, several key regulators of cell cycle progression, including AKT1, AKT2, mTOR, CDK4, CDK6, and CDC45, were among the most significantly suppressed proteins (Fig. 1D, F–K). In line with these proteomic findings, flow cytometric analysis demonstrated that EB exposure resulted in significant G2/M phase accumulation, with a proportional decline in G0/G1 and S phase cell populations (Fig. 1L and Fig. S1C). Together, these results indicate that EB mediates robust cytotoxicity in HL-60 leukemia cells through the induction of apoptosis and G2/M phase cell cycle arrest.

### Chemoproteomic profiling identifies PSMD14 as the primary covalent target of EB

**Fig. 2:**
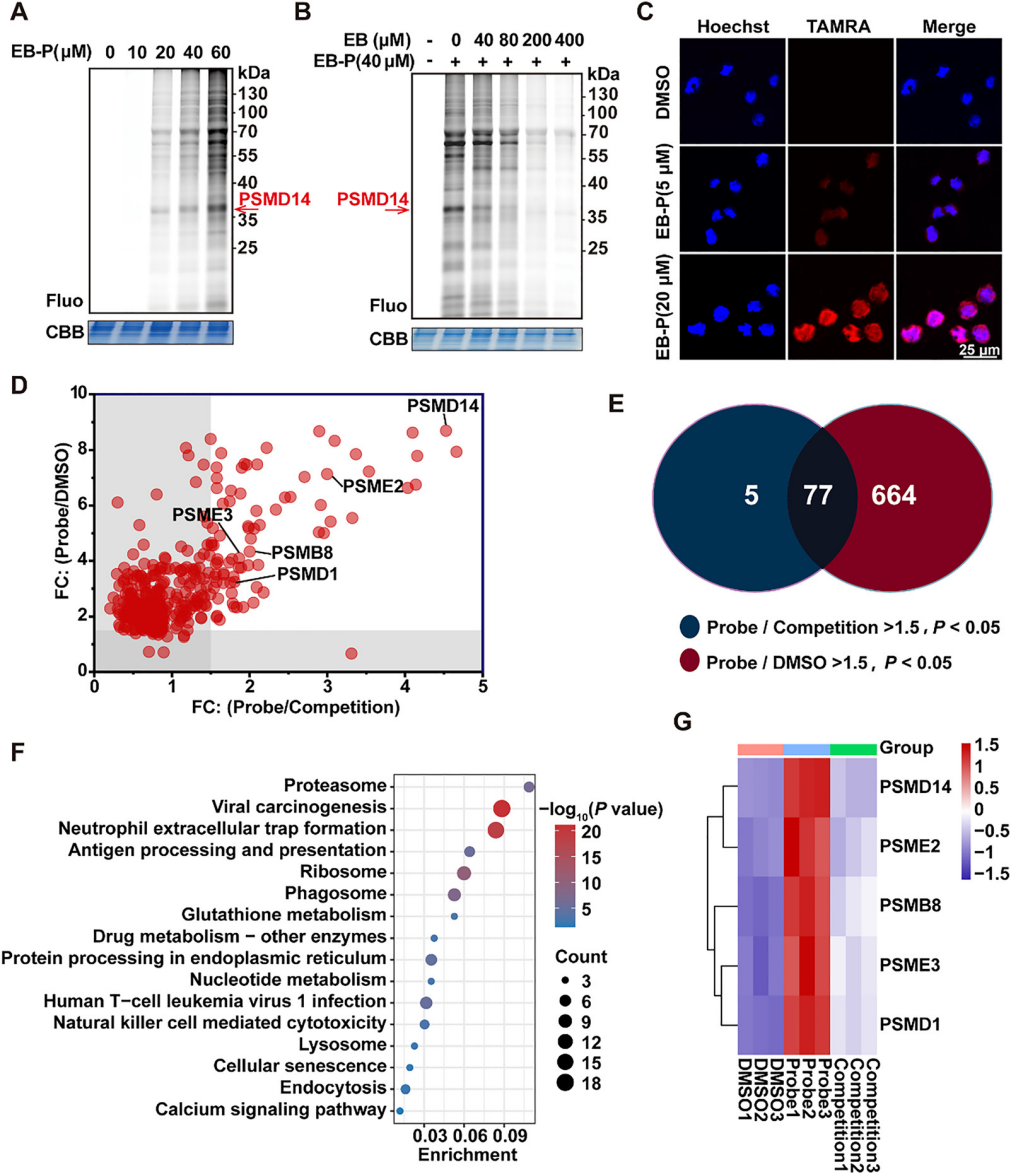
(A) *In situ* fluorescence labeling of EB-P at varying concentrations in HL-60 cells. (B) *In situ* fluorescence labeling of EB-P in the absence or presence of excess unlabeled EB as a competition control. (C) Confocal cellular imaging of EB-P in HL-60 cells (scale bar = 25 μm). (D) Scatter plots showing the ratios of target proteins between the competition and probe groups (*p* < 0.05). (E) Venn diagram illustrating the overlap of target proteins identified in the probe and competition groups using a cutoff of FC > 1.5 and *p* < 0.05. (F) KEGG pathway enrichment analysis of the overlapping target proteins. (G) Heatmap showing the relative abundance of proteasome-related proteins across control, probe, and competition groups.

In order to clarify the molecular mechanisms responsible for cell cycle arrest and apoptosis triggered by EB exposure, we employed an activity-based protein profiling (ABPP) strategy for target deconvolution. Leveraging EB's previously reported covalent binding capability,^9^ we designed and synthesized a clickable, alkyne-tagged EB-P *via* a concise one-step synthetic route (Fig. 1M). Importantly, EB-P retained potent anti-proliferative activity against HL-60 cells, comparable to that of the parent compound (Fig. 1N), thereby validating its functional relevance. *In situ* fluorescence labeling of live HL-60 cells using EB-P revealed dose-dependent protein labeling, consistent with direct covalent target engagement (Fig. 2A). This labeling was effectively competed by pre-treatment with excess native EB in a dose-dependent manner (Fig. 2B), confirming the specificity of binding and demonstrating that EB-P and EB share the same target profile. Confocal imaging further revealed efficient intracellular localization of EB-P to both the cytoplasm and nucleus (Fig. 2C), consistent with its biological activity.

For comprehensive target identification, live HL-60 cells were pre-incubated with either EB (200 μM) or DMSO, followed by labeling with EB-P (40 μM). EB-P-bound proteins were conjugated to biotin-azide *via* copper-catalyzed azide–alkyne cycloaddition (CuAAC), enriched using NeutrAvidin agarose, and analyzed by liquid chromatography-tandem mass spectrometry (LC-MS/MS). To ensure high-confidence target identification, we applied stringent filtering criteria: proteins showing significant enrichment in the probe-treated group compared to control (EB-P/DMSO, FC > 1.5, *p* < 0.05; 741 proteins) and significant reduction in the competition group (EB-P *vs.* EB-P + EB, FC > 1.5, *p* < 0.05; 82 proteins) were retained (Fig. 2D and E). Intersection analysis identified 77 high-confidence covalent targets of EB (Fig. 2E). Kyoto Encyclopedia of Genes and Genomes (KEGG) pathway analysis of these target proteins revealed the proteasome pathway as the most significantly enriched (Fig. 2F). Heatmap visualization further confirmed strong enrichment of multiple proteasome subunits, with PSMD14 emerging as a prominently labeled target (Fig. 2G). These results strongly implicate the proteasome, specifically PSMD14, as the primary functional target mediating EB's anti-leukemic activity.

### EB covalently binds and inhibits the DUB activity of PSMD14

**Fig. 3:**
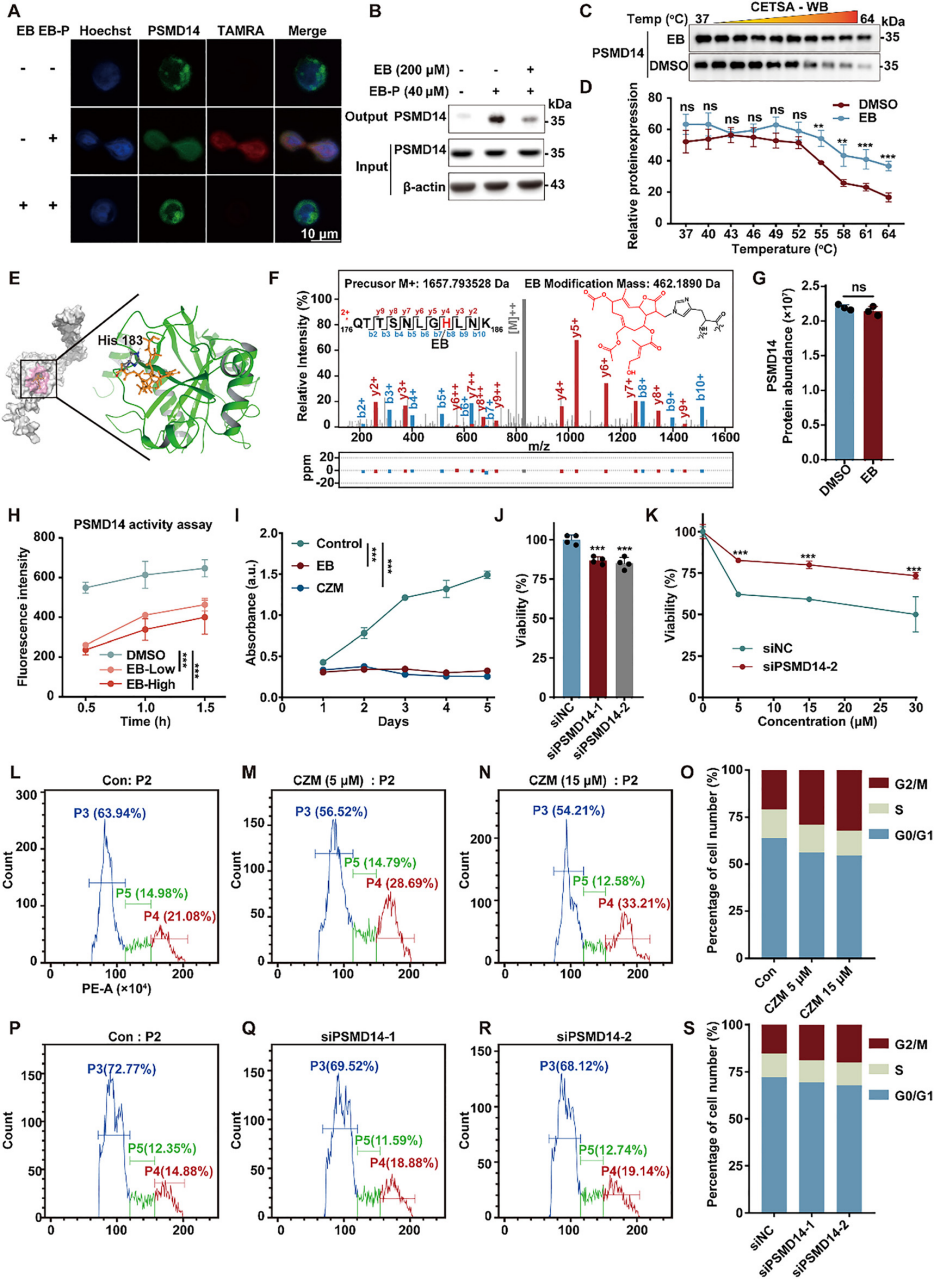
(A) Co-localization of PSMD14 (green) and EB-P (red) in HL-60 cells confirmed by immunofluorescence staining (scale bar = 10 μm). (B) Pull-down assay using EB-P, followed by Western blotting, confirming that EB binds to PSMD14 *in situ*. (C and D) Cellular thermal shift assay (CETSA)-WB indicating the direct interaction between EB and PSMD14. (E, Left) Structural model of the predicted PSMD14–EB complex. The protein is shown as a gray cartoon, and EB is depicted in orange sticks. Residues forming the predicted binding pocket are highlighted. (E, Right) Detailed view of the predicted binding interface. The key residue His183 of PSMD14 is represented as sticks and is labeled. (F) Mapping of the EB binding site on recombinant human PSMD14 by liquid chromatography-tandem mass spectrometry (LC-MS/MS). (G) Western blot showing PSMD14 protein levels in HL-60 cells incubated with or without EB (15 μM). (H) *In vitro* activity assay showing that EB (100 μM or 200 μM) significantly inhibits PSMD14 enzymatic activity. (I) Both EB (15 μM) and CZM (40 μM, a known PSMD14 inhibitor) markedly inhibited HL-60 cell proliferation. (J) Cell viability was assessed by CCK-8 assay following PSMD14 knockdown. (K) HL-60 cells transfected with siPSMD14-2 or siNC were treated with different concentrations of EB, and cell viability was measured by CCK-8 assay. (L–O) Cell cycle distribution of HL-60 cells treated with CZM. (P–S) PSMD14 knockdown significantly altered cell cycle progression in HL-60 cells. **P < 0.01, ***P < 0.001, ns = not significant.

Given PSMD14's top ranking in both probe enrichment and competitive displacement efficiency (Fig. 2D), it was prioritized for functional validation. Immunofluorescence staining demonstrated marked co-localization of EB-P labeling with endogenous PSMD14 in both the cytoplasmic and nuclear compartments of HL-60 cells. This co-localization was markedly diminished by pre-incubation with excess native EB (Fig. 3A), supporting the specificity of EB-P binding to PSMD14. Additionally, EB-P successfully pulled down PSMD14 from HL-60 cell lysates, and this interaction was competitively inhibited by unmodified EB (Fig. 3B). Cellular thermal shift assay (CETSA) further demonstrated a significant increase in the thermal stability of PSMD14 upon EB incubation (Fig. 3C and D), indicating direct binding and protein stabilization. Furthermore, *in silico* molecular docking analysis identified His183 as a critical residue involved in PSMD14 binding to EB (Fig. 3E). To further confirm the covalent nature of this interaction, tandem mass spectrometry was used to analyze the mass shift of peptides after recombinant human PSMD14 was incubated with EB. The results revealed the formation of an EB adduct at His183 (Fig. 3F), indicating a covalent bond between the histidine residue and the reactive α-methylene-γ-lactone moiety of EB, a modification consistent with previous reports.^10,11^ Importantly, EB did not affect PSMD14 protein abundance (Fig. 3G), suggesting that its functional effects arise from post-translational modulation rather than changes in expression. As PSMD14 functions as a JAMM family DUB within the 19S regulatory particle of the proteasome, we next evaluated the impact of EB on its enzymatic activity. EB inhibited the deubiquitylating activity of the purified 26S proteasome in a concentration-dependent manner (Fig. 3H and Fig. S2A–C), closely recapitulating the effects of the known PSMD14 inhibitor Capzimin (CZM), thereby confirming EB's role as a covalent PSMD14 DUB inhibitor.

To investigate the functional consequences of PSMD14 inhibition, we first compared the effects of EB with those of CZM, a well-characterized and selective PSMD14 inhibitor.^13^ CZM treatment closely mirrored EB's anti-proliferative effects in HL-60 cells over a 5-day period (Fig. 3I). Furthermore, siRNA-mediated knockdown of PSMD14 significantly impaired HL-60 cell proliferation (Fig. 3J). Crucially, PSMD14 silencing markedly attenuated the cytotoxic effects of EB (Fig. 3K and Fig. S2D and E), confirming that PSMD14 is the primary functional target responsible for EB-induced lethality in leukemia cells. Given the observed G2/M arrest induced by EB, we next examined whether modulation of PSMD14 produced similar effects on cell cycle progression. Treatment with CZM significantly reduced the proportion of cells in the G0/G1 and S phases, while increasing accumulation in the G2/M phase (Fig. 3L–O), thus phenocopying the effects of EB. Similarly, genetic depletion of PSMD14 reproduced this G2/M cell cycle arrest phenotype (Fig. 3P–S). Together, these findings demonstrate that PSMD14 activity is crucial for maintaining normal cell cycle progression in HL-60 cells and establish PSMD14 as the critical target mediating EB's anti-leukemic activity.

### EB promotes degradation of AKT1 and CDK4 oncoproteins *via* PSMD14 inhibition

**Fig. 4:**
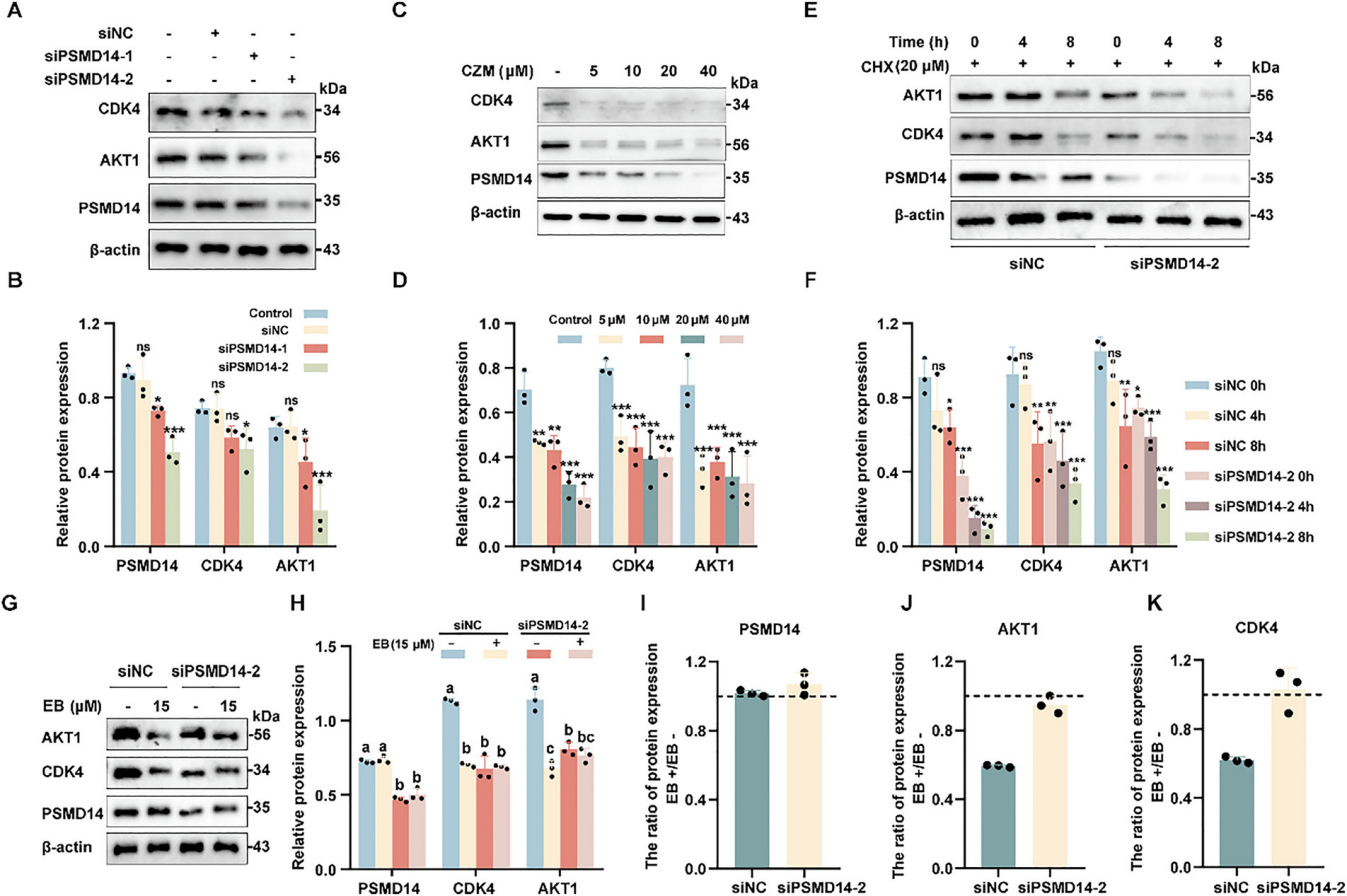
(A and B) WB analysis of AKT1 and CDK4 protein expression following PSMD14 knockdown in HL-60 cells. (C and D) WB analysis of PSMD14, AKT1, and CDK4 in HL-60 cells treated with the PSMD14 inhibitor CZM. (E and F) Expression of PSMD14, AKT1, and CDK4 after treatment with cycloheximide (CHX, 20 μM), analyzed by Western blot. (G and H) WB analysis of PSMD14, AKT1, and CDK4 in HL-60 cells transfected with siPSMD14-2 and subsequently treated with EB; siNC was used as the negative control. (I–K) Quantification of PSMD14 (I), AKT1 (J), and CDK4 (K) protein levels with or without EB treatment. *P < 0.05, **P < 0.01, ***P < 0.001, ns = not significant. Identical letters indicate no statistically significant difference, while different letters indicate *P* < 0.05.

Our initial proteomic analysis confirmed that EB exposure downregulated the expression levels of key oncoproteins AKT1 and CDK4 (Fig. 1D). Given the established role of PSMD14 in stabilizing specific substrates *via* deubiquitylation, we hypothesized that EB induces the degradation of these proteins through inhibition of PSMD14 activity. Supporting this hypothesis, siRNA-mediated knockdown of PSMD14 alone resulted in a marked reduction in AKT1 and CDK4 protein levels (Fig. 4A and B). Similarly, pharmacological inhibition of PSMD14 using CZM led to a dose-dependent decrease in the levels of AKT1, CDK4, and PSMD14 itself (Fig. 4C and D). To directly assess the impact of PSMD14 inhibition on protein stability, we performed cycloheximide (CHX) chase assays following PSMD14 silencing. The degradation kinetics of both AKT1 and CDK4 were significantly accelerated in PSMD14-deficient cells (Fig. 4E and F), confirming the role of PSMD14 in maintaining the stability of these oncoproteins. To further determine whether EB-induced degradation of AKT1 and CDK4 is dependent on PSMD14 inhibition, we compared protein levels in EB-treated cells with and without PSMD14 knockdown. While EB effectively suppressed AKT1 and CDK4 expression in control HL-60 cells (Fig. 4G and H), this effect was completely abolished in PSMD14-depleted cells (Fig. 4I–K). Together, these data establish that EB induces AKT1 and CDK4 degradation through specific inhibition of PSMD14-dependent deubiquitination and stabilization, further substantiating PSMD14 as a critical mediator of EB's anti-leukemic activity.

## Discussion

Although APL is now considered highly curable with ATRA and ATO–based regimens, treatment is still associated with significant toxicities, including life-threatening coagulopathy, neuro- and cardiotoxicity, and an increased risk of secondary malignancies.^3,5,6^ These limitations highlight the urgent need for safer, mechanistically distinct therapeutic strategies. In this study, we identify the natural sesquiterpene lactone EB as a potent anti-leukemic compound active against APL-derived HL-60 cells. Using an integrated chemoproteomic and functional validation approach, we establish PSMD14, a JAMM family DUB, as the primary covalent intracellular target of EB. Importantly, we show that EB binding inhibits the deubiquitylase activity of PSMD14, leading to the destabilization and proteasomal degradation of key oncogenic substrates, namely AKT1 and CDK4, whose stabilization by PSMD14 is essential for HL-60 cell proliferation. This mechanism results in G2/M cell cycle arrest and apoptotic cell death. Collectively, our findings reveal a previously unrecognized PSMD14–AKT1/CDK4 stabilization axis that is critical for leukemia cell survival. Moreover, this study positions EB as both a mechanistic probe for interrogating deubiquitination pathways and a promising lead compound for the development of covalent DUB inhibitors as anticancer therapeutics.

As a JAMM family DUB embedded in the 19S regulatory particle of the 26S proteasome, PSMD14 governs protein fate by either facilitating substrate degradation, *via* removal of proximal ubiquitin chains to enable translocation into the 20S catalytic core, or rescuing ubiquitinated proteins from degradation through deubiquitylation, thus stabilizing them.^14,15^ This functional duality is frequently co-opted in cancer. PSMD14 is notably overexpressed in a wide range of malignancies,^16–18^ where it functions as an oncogenic DUB by selectively stabilizing pro-survival and proliferative proteins while promoting the turnover of tumor suppressors. For instance, PSMD14 has been reported to stabilize transcription factors such as snail family transcriptional repressor 1 (SNAIL) to promote metastasis,^19^ as well as oncoproteins like Myc proto-oncogene protein (MYC) and activin receptor type-1 (ALK2) to sustain proliferation.^18,20^ This dysregulation contributes to tumor progression, therapeutic resistance, and poor clinical outcomes.^15^ Thus, translational research on PSMD14 is both highly promising and urgently needed. Currently, known inhibitors of PSMD14 include 8-Mercapto-*N*-[(tetrahydro-3-furanyl)methyl]-4-quinolinecarboxamide, *o*-phenanthroline (OPA), CZM, thiolutin (THL), and epidithiodiketopiperazines.^13,21–24^ These compounds inhibit the deubiquitinating enzyme activity of PSMD14 and have shown therapeutic potential in various cancers, including multiple myeloma.^25^ However, among these, only 8-Mercapto-*N*-[(tetrahydro-3-furanyl)methyl]-4-quinolinecarboxamide has advanced to early clinical trials,^21^ while the others remain at the preclinical stage. Our identification of EB as a covalent inhibitor of PSMD14 is therefore of considerable mechanistic and translational importance. Featuring a reactive α-methylene-γ-lactone moiety, a characteristic structural motif of sesquiterpene lactones, EB represents a novel chemotype for selectively targeting PSMD14. Accordingly, EB not only serves as a valuable chemical probe for elucidating the oncogenic roles of PSMD14 but also offers a promising lead compound for the development of selective covalent DUB inhibitors with potential applications in cancer therapy.

The oncoproteins AKT1 and CDK4/6, whose degradation is facilitated by EB through PSMD14 inhibition, represent central nodes in oncogenic signaling networks. AKT1, a pivotal regulator of the PI3K/AKT/mTOR pathway, orchestrates multiple cellular processes including survival, proliferation, metabolism, and apoptosis evasion.^26^ CDK4/6, when complexed with cyclin D, phosphorylates the retinoblastoma protein (Rb) to drive the G1/S phase cell cycle transition, constituting a critical dependency in numerous cancers.^27^ Dysregulation of these proteins is a hallmark of leukemogenesis and various other malignancies.^27–29^ Our findings uncover a previously unappreciated regulatory mechanism whereby PSMD14 stabilizes both AKT1 and CDK4 by antagonizing their ubiquitin-mediated proteasomal degradation. Inhibition of PSMD14's DUB activity, either pharmacologically by EB or CZM or *via* genetic knockdown, abolishes this protective effect, thereby permitting the ubiquitination-dependent degradation of AKT1 and CDK4. This direct mechanistic link between PSMD14 activity and the stabilization of key oncogenic drivers reveals a novel therapeutic vulnerability in leukemia cells that can be exploited by targeted DUB inhibition.

While our study establishes PSMD14 as the principal functional target underlying EB's anti-leukemic activity, several key mechanistic and translational questions remain to be addressed. First, the promising *in vitro* anti-leukemic effects observed in HL-60 cells require validation in more physiologically relevant *in vivo* models. Comprehensive assessment of EB's pharmacokinetics, pharmacodynamics, efficacy, and safety in established APL animal models, such as PML-RARα-driven genetically engineered models or patient-derived xenografts, is necessary to evaluate its translational potential and guide preclinical development. Secondly, although chemoproteomic profiling identified 77 candidate EB-interacting proteins, PSMD14 emerged as the dominant mediator of cytotoxicity; nevertheless, the roles of these secondary targets in EB's overall pharmacological effects, including potential synergistic anti-leukemic actions or off-target toxicities, remain to be defined. Systematic characterization of these additional targets will deepen mechanistic insight and facilitate the refinement of EB as a targeted therapeutic. Finally, although we identified His183 as the specific site of EB's covalent binding to PSMD14, the detailed inhibition mechanism and its rational basis remain to be elucidated. Such understanding is critical for the structure-guided optimization of EB derivatives with improved potency and selectivity. Addressing these critical issues will be essential to advance EB or optimized analogues toward clinical evaluation as innovative PSMD14-targeted therapies for leukemia.

## Conclusion

In summary, this study identifies EB as a potent covalent inhibitor of the DUB PSMD14, revealing a novel molecular mechanism by which EB exerts anti-leukemic effects in APL-derived HL-60 cells. By targeting PSMD14, EB disrupts the stabilization of critical oncoproteins AKT1 and CDK4, thereby inducing G2/M cell cycle arrest and apoptosis. Our integrated chemoproteomic and functional analyses not only establish PSMD14 as a promising therapeutic target in leukemia but also position EB as a valuable chemical probe and lead compound for the development of selective covalent inhibitors against this oncogenic DUB. Future efforts focusing on elucidating the precise covalent binding mechanism, *in vivo* validation, and comprehensive profiling of secondary targets will be pivotal for translating EB into clinically viable therapies. Collectively, these findings provide a strong foundation for advancing PSMD14-targeted strategies in leukemia treatment.

## Materials and methods

### General

Antibodies used for immunoblotting (IB) and immunofluorescence (IF) experiments: AKT1 Rabbit mAb, A17909 (IB, 1 : 1000); PSMD14 Rabbit mAb, A9608 (IB, 1 : 1000); β-Actin Rabbit mAb, AC026 (IB, 1 : 50 000); FITC Goat Anti-Rabbit IgG, AS011 (IB, 1 : 200) were procured from ABclonal (Wuhan, China). Goat Anti-Rabbit IgG, 511203 (IB, 1 : 5000) was purchased from ZENBIO (Chengdu, China). Anti-CDK4 antibody, ab199728 (IB, 1 : 2000) was obtained from Abcam (Shanghai, China).

All commercially available solvents and reagents were used without further purification. Z-VAD-FMK and CZM were procured from TargetMol Chemicals Inc. (Boston, MA, USA). EB was acquired from Shanghai Macklin Biochemical Technology Co., Ltd (Shanghai, China). Cell Cycle and Apoptosis Analysis Kit, Hoechst dye, Annexin V-FITC Apoptosis Detection Kit, NP-40, Mitochondrial Membrane Potential Assay Kit with JC-1 were acquired from Beyotime Biotech Inc. (Shanghai, China). Tris[(1-benzyl-1*H*-1,2,3-triazol-4-yl) methyl] amine (TBTA), triethylammonium bicarbonate (TEAB), dithiothreitol (DTT), CuSO4, Biotin-azide, iodoacetamide (IAA), Rhodamine-azide, Tris(2-carboxyethyl) phosphine (TCEP) were acquired from Sigma (St. Louis, MO, USA). High-capacity neutravidin agarose resin, Acetonitrile (ACN), RPMI 1640, Formic acid (FA), TMT 10plex™ reagent, Halt™ protease inhibitor cocktail (PI), penicillin–streptomycin (PS) and Trypsin were procured from Thermo Fisher Scientific (Waltham, MA, USA). 26S proteasome (RP10136TLQ) was purchased from ABclonal (Wuhan, China). Ub-AMC substrate was obtained from ks-vpeptide (Hefei, China). Epoxomicin was procured from MedChemExpress (Shanghai, China). ATP were purchased from Aladdin (Shanghai, China). Fetal bovine serum (FBS) are mailed from Corning (NY, USA). Lipofectamine 2000 Transfection Reagent was from Invitrogen.

### Cell culture

HL-60 cells were procured from the Chinese Academy of Medical Sciences (Beijing, China). Cells were cultured in a humidified incubator aseptically (37 °C, 5% CO_2_). RPMI 1640 medium (supplementing 20% FBS, 1% PS) was applied for cells culture. Experiments were performed only when cell viability exceeded 95%.

### Cytotoxicity assays

Cell Counting Kit-8 (Meilunbio, China) was employed for the cytotoxicity assays according to the manual. Briefly, HL-60 cells were cultured using 96-well microplates for 24 h. The culture media containing different concentrations of EB or EB-P were added for a 24 h incubation period after the removal of the old media. After that, each well received an addition of 10 µL Cell Counting Kit solution. For apoptosis reversal assays, Z-VAD-FMK was administered at 40 µM to the relevant groups. Following an additional 2 h incubation period in the incubator, cellular absorbance at 450 nm was measured using the EnVision 2105 Multimode Plate Reader (PerkinElmer, USA).

### Cell cycle, apoptosis analysis and mitochondrial membrane potential determination

These assays were performed using the Kits, following the manufacturers’ protocols. During the exponential growth phase, HL-60 cells were exposed to either EB or CZM for specified durations. Following harvesting, cells were stained following the manufacturer's protocols and subjected to flow cytometric analysis using a CytoFLEX analyzer (Beckman Coulter, Brea, CA, USA). Data acquisition and analysis, including quantification of DNA content, were conducted using FlowJo software (BD Biosciences, San Jose, CA, USA).

### Proteomics and data analysis

Prior to lysis, cells were pre-incubated with either EB (15 µM) or DMSO (0.4%, as negative control) and subsequently lysed in 0.1% (v/v) Triton X-100 buffer containing 1× PI. Lysates were centrifuged to collect total proteins. Protein reduction and alkylation were performed using DTT and IAA, respectively. Protein precipitation was carried out using 1 mL of pre-chilled acetone (−20 °C, 16 h), followed by centrifugation. The protein pellets were dissolved in TEAB buffer (100 mM). Digestion was carried out with trypsin (12.5 ng µL^−1^) at 37 °C for 16 h. The digested peptides were collected by centrifugation (10 000 × *g*, 10 min, 4 °C) and transferred through a filter-spin column. Digestion was stopped by adding 0.1% FA. Peptides were desalted using C18 columns (Waters, USA) and concentrated by lyophilization. Samples were reconstituted in 60 µL of solvent (1% ACN, 0.1% FA), and 20 µL was subjected to LC-MS/MS analysis using a Thermo Orbitrap Fusion Lumos system (Thermo Fisher Scientific, USA). Differentially expressed proteins (DEPs) were identified using criteria of absolute FC > 1.5 and *p* < 0.05. GO analysis was conducted to annotate and visualize the biological pathways linked to these DEPs.

### Fluorescence labelling of HL-60 cells

The fluorescence labeling protocol was adapted slightly from a previously described method.^30^ HL-60 cells were exposed to EB-P or DMSO for 2 h (37 °C, 5% CO_2_). For competition assays, cells were first treated with an excess of EB for 1 h before EB-P exposure. Following incubation, cells were lysed and the protein concentrations were determined. Equal protein quantities from each sample were applied for fluorescence labeling. The lysates were treated by sequential addition of the following reagents for each reaction: TBTA ligand (100 μM), rhodamine-azide (50 μM), CuSO_4_ (1 mM), and TCEP (1 mM). Following a 2 h incubation with shaking at 37 °C, proteins were precipitated by adding chilled acetone. The resulting pellets were air-dried, dissolved in 1× SDS loading buffer, and boiled at 95 °C for 10 min. Then, each sample (15 μL) was resolved on 10% polyacrylamide gels using SDS–PAGE. After electrophoresis separation, proteins were visualized by fluorescence scanning (Sapphire Biomolecular Imager, Azure Biosystems) and verified by Coomassie Brilliant Blue staining for loading normalization.

### Cellular imaging

Prior to fixation with 4% paraformaldehyde (15 min), HL-60 cells were treated with EB-P either with or without EB competition. Following centrifugation, the cell pellet was PBS-washed and transferred to poly-l-lysine-coated slides for mounting. Cells were permeabilized with 0.2% Triton X-100 for 15 min, followed by the addition of the click chemistry reaction mixture as described above. Subsequently, cells were stained with Hoechst nuclear dye for 20 min at room temperature and washed twice with PBS. Fluorescence images were acquired using confocal microscopy. For fluorescence colocalization assays, after the click chemistry reaction and PBS washes, cells were incubated with primary antibody against PSMD14 (1 : 200) at 4 °C for 12 h, followed by two PBS washes. Cells were then stained with fluorescently labeled secondary antibody (1 : 500) and Hoechst (1 : 500), washed twice with PBS, and imaged by laser scanning confocal microscopy.

### Target identification by chemoproteomics

HL-60 cells were incubated with EB-P (40 μM) for 2 h at 37 °C. For competition assays, cells were pre-incubated with excess EB (5×) for 1 h prior to EB-P treatment. Cells treated with 0.4% DMSO served as negative controls. Cell proteins were isolated and quantified as described previously. Subsequently, proteins were conjugated to biotin tag through click chemistry reaction. For each sample, TCEP (1 mM), CuSO_4_ (1 mM), biotin–azide (50 μM) and TBTA ligand (100 μM) were sequentially added, and the reaction mixtures were incubated at 37 °C with shaking for 2 h. Acetone-precipitated click-labeled proteins were completely solubilized in 5 mL of 0.1% SDS solution, then incubated with high-capacity neutravidin agarose beads for 4 h at 26 °C under gentle mixing. Beads were sequentially washed with SDS (1%), SDS (0.1%) and urea (6 M) solution, with three washes per solution before proceeding to the next. Afterwards, beads were resuspended in 0.5 mL of 6 M urea. Enriched proteins underwent reduction and alkylation using DTT (10 mM) and IAA (20 mM), respectively. Trypsin (12.5 ng μL^−1^) was applied to initiate protein digestion, which was carried out at 37 °C for 17 h. Peptides were collected and digestion was quenched by addition of FA (0.1%). The isolated peptides were desalted using a C18 column and lyophilized. Subsequently, the dried peptides were dissolved in TEAB (100 mM) and labeled with a TMT 10plex™ Mass Tag reagent. Labeled peptides were pooled, desalted again with a C18 column, and lyophilized. For LC-MS/MS analysis, samples were reconstituted in 100 μL of dilution buffer (0.1% FA, 1% ACN) and stored at 4 °C until analysis. For verification of pull-down target proteins, beads were washed after incubation, followed by addition of 1× SDS loading buffer to dissolve the pellet. Samples were boiled at 95 °C for 10 min and analyzed by Western blotting (WB).

### WB assays

HL-60 cells from various experiments were lysed to extract total proteins. Proteins were separated by SDS-PAGE according to their molecular weights and subsequently transferred onto PVDF membranes *via* electroblotting. Membranes were incubated with the appropriate primary antibodies specific to the target proteins at 4 °C for 10 h. Post-TBST washing (three times), the membranes were exposed to the relevant secondary antibodies for 2 h with mild agitation. Protein bands were visualized using enhanced chemiluminescence (ECL) on an Azure C400 imaging system (Azure Biosystems, USA). The protein bands were analyzed densitometrically with ImageJ software (National Institutes of Health, USA).

### CETSA

To further validate PSMD14 as the direct target of EB, CETSA coupled with WB (CETSA-WB) was performed. Protein extracts were prepared from HL-60 cells, and equivalent protein quantities were treated with EB or DMSO at room temperature before heat exposure. Aliquots were distributed into PCR tubes and processed through a temperature gradient protocol using an Applied Biosystems thermal cycler (Thermo Scientific, USA). Following thermal treatment at different temperatures, the samples were pelleted by centrifugation (16 000 × *g*, 4 °C, 10 min). The clarified supernatants were combined with SDS loading buffer, heat-denatured (95 °C, 5 min), then subjected to western blot analysis. Quantitative assessment of band intensities was performed with ImageJ.

### Computational modeling

The full-length three-dimensional structure of PSMD14 (residues 1–310) was predicted using the AlphaFold server.^31^ Molecular docking of EB to PSMD14 was performed using the Maestro software on the Schrödinger platform, with EB ligand preparation and optimization conducted *via* the LigPrep and Glide program was employed to dock EB into the binding pocket of PSMD14.^32^

### Binding site identification

To identify the direct binding site of EB on PSMD14, 40 μg of recombinant human PSMD14 protein (Signalchem Lifesciences) was incubated with 40 μM EB for 2 h at 37 °C. Subsequently, the samples were reduced with 10 mM DTT and alkylated with 20 mM IAA for 30 min each. To remove excess compound and reagents, proteins were precipitated using six volumes of pre-cooled acetone. Finally, the protein pellets were digested with 1 μg trypsin, and the resulting peptides were desalted using a C18 column prior to analysis by LC-MS/MS.

### *In vitro* PSMD14 activity assay

PSMD14 activity was assessed using fluorescence polarization measurements in black 96-well microplates. The reaction buffer contained 50 μM ATP, 50 mM Tris–HCl (pH 7.5), 0.01% NP-40, 1 mM MgCl_2_ and 1 mM DTT. The 26S proteasome was pre-treated with epoxomicin (100 μM) for 1 h (26 °C), followed by incubation with either 0.4% DMSO (negative control) or EB at 200 μM or 400 μM for 1 h. Subsequently, equal volumes of each sample were mixed with the reaction buffer containing Ub-AMC (500 nM, dissolved in DMSO), transferred to the 96-well plates, and incubated for 30 min (37 °C). Fluorescence polarization was measured at 30 °C using an EnVision 2105 Multimode Plate Reader.

### Transfection assay

Duplex small interfering RNAs (siRNAs) targeting PSMD14 and a negative control (siNC) were chemically synthesized by GENERAY (Shanghai, China). The siRNA sequences were as follows: siPSMD14-1 (5′-GGTCTTAGGACATGAACCA-3′), siPSMD14-2 (5′-GTGATTGATGTGTTTGCTA-3′), and siNC (5′-UUCUCCGAACGUGUCACGUTT-3′). HL-60 cells were transfected with either PSMD14-targeting siRNAs or siNC using Lipofectamine 2000 according to the manufacturer's protocol. After 48 h of culture, cell viability assays were performed, and protein expression levels were assessed.

### Statistical analysis

The software of GraphPad Prism 8.0 (GraphPad software, Inc., USA) was used in this study to calculate statistical significance. Unless otherwise stated, all values were expressed as mean ± standard deviation (SD). Comparisons between two groups were conducted using a two-tailed unpaired Student's *t*-test, while multiple group comparisons were analyzed using one-way or two-way analysis of variance (ANOVA), as appropriate. Results with *P* value less than 0.05 were recognized as statistically significant.

## Author contributions

H. T., J. W., and C. X. conceived the project and supervised the study. Z. C., L. X., H. C., and T. F. designed and performed all experiments with the help from the other authors. X. H., Y. K. W., Q. S., and J. Z. contributed to the acquisition, analysis, and interpretation of the data. H. T., Z. C., and L. X. wrote the paper and revised the manuscript. All authors have read and approve the final manuscript.

## Conflicts of interest

The authors declare no conflicts of interest.

## Supplementary Material

CB-OLF-D5CB00197H-s001

## Data Availability

The datasets used and/or analyzed during the present study are available from the corresponding author on reasonable request. The data for flow cytometry analysis, enzyme activity of PSMD14, and original Western blot/gel results is included in supplementary information. Supplementary information (SI) is available. See DOI: https://doi.org/10.1039/d5cb00197h.
