# Supplementary material for: Covalent targeting of PSMD14 by Eupalinolide B induces oncoprotein degradation and apoptosis in acute promyelocytic leukemia cells
Source: RSC Chem Biol. 2026 Feb 4;7(3):433–43. doi: 10.1039/d5cb00197h (PMC12869703; doi:10.1039/d5cb00197h)

Supplementary Information (SI) for RSC Chemical Biology.
This journal is © The Royal Society of Chemistry 2026

Supporting Information

Covalent Targeting of PSMD14 by Eupalinolide B Induces Oncoprotein Degradation and Apoptosis in Acute Promyelocytic Leukemia Cells

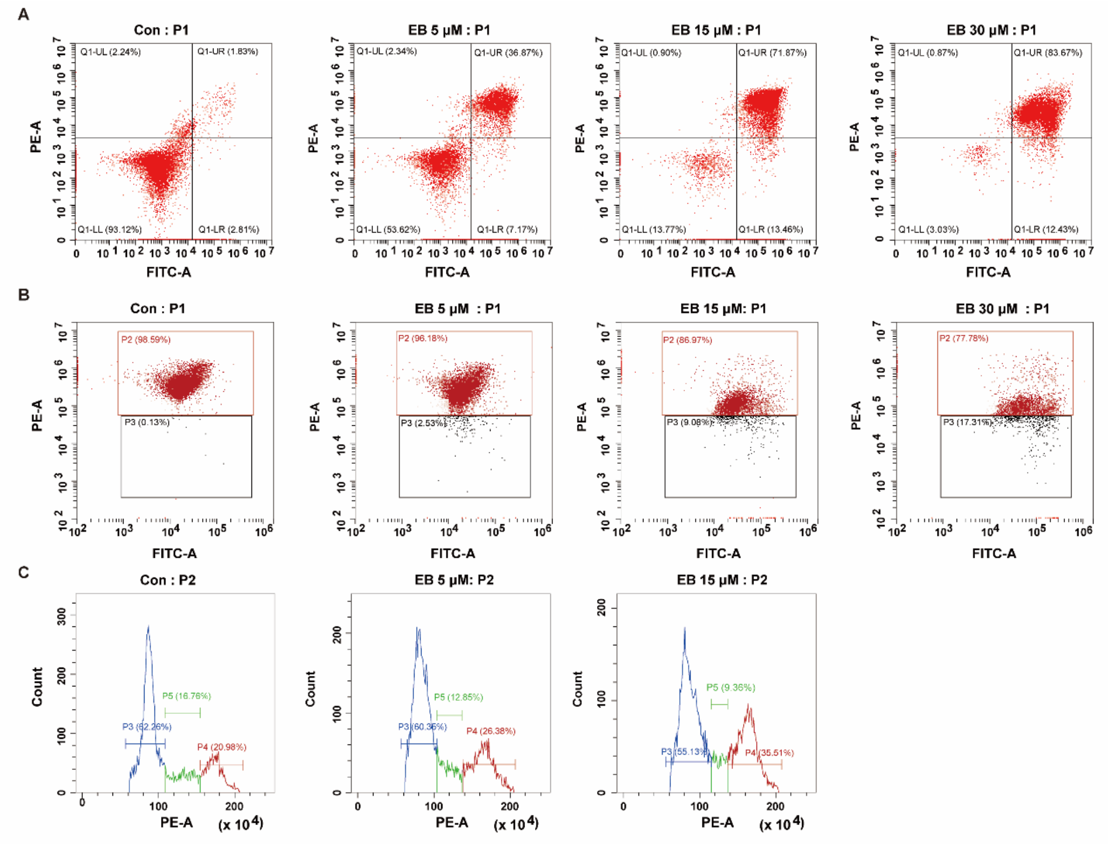

Figure S1. (A) EB induced apoptosis of HL-60 cells in a dose dependent manner. (B) EB reduced mitochondrial membrane potential in HL-60 cells in a dose dependent manner. (C) EB significantly affected the cell cycle progression of HL-60 cells.

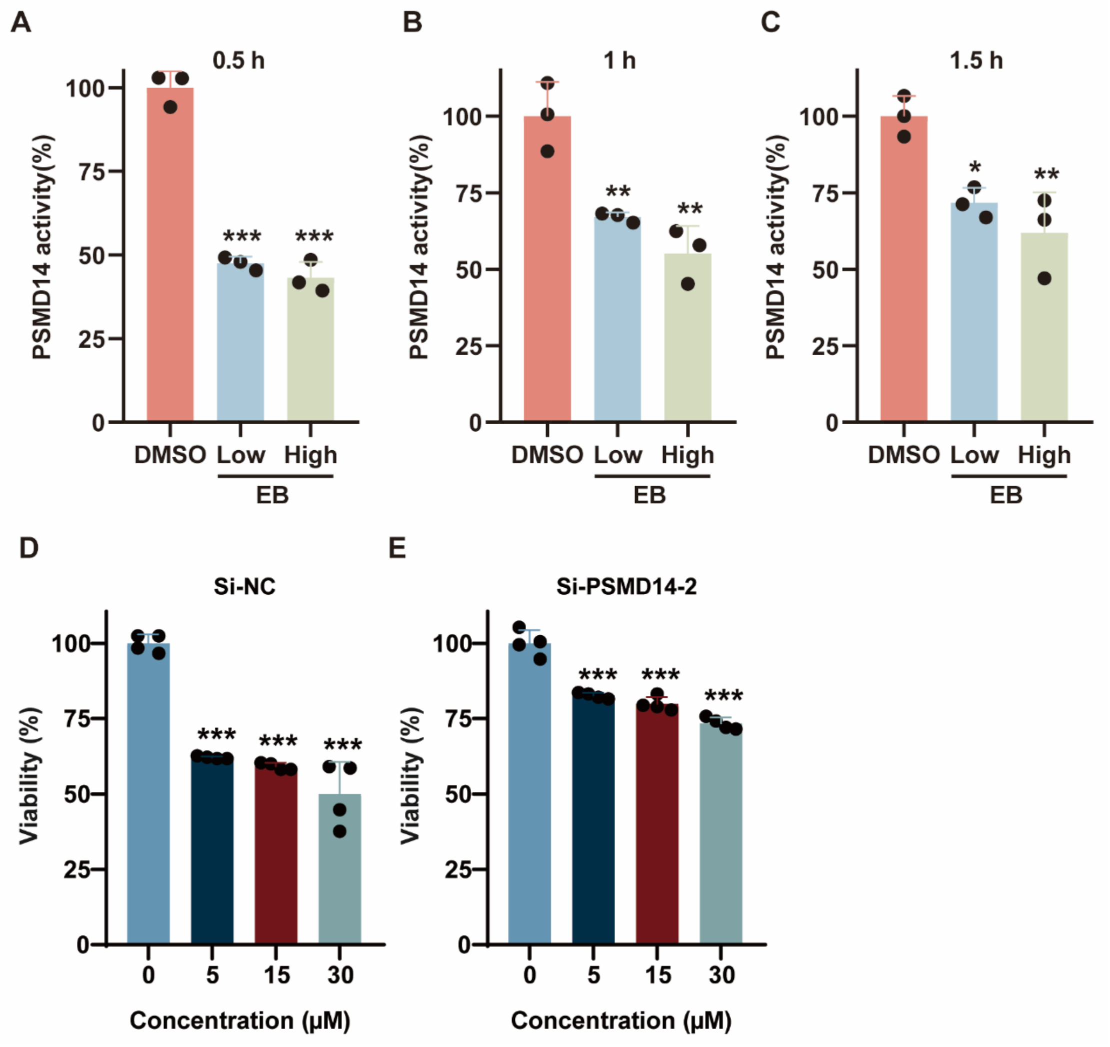

Figure S2. (A-C) The enzyme activity of PSMD14 was significantly inhibited by EB. (D) EB had a strong inhibitory effect on normal HL-60 cells. (E) PSMD14 knockdown alleviated the inhibitory effect of EB on HL-60 cells.

All raw images for western blot/gel results

Figure 3B PSMD14 (Output)

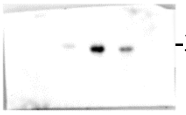

Figure 3B PSMD14 (Input)

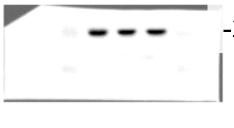

Figure 3B β-actin

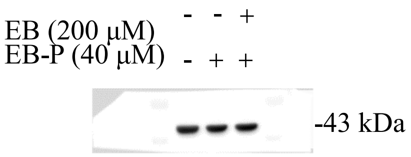

Figure 3C EB

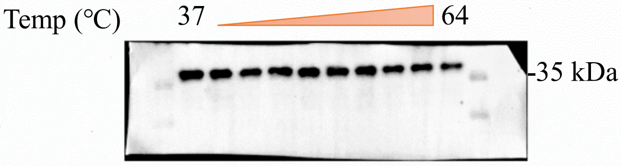

Figure 3C DMSO

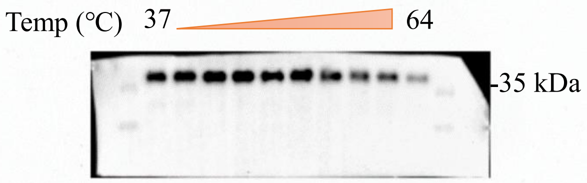

Figure 4A β-actin

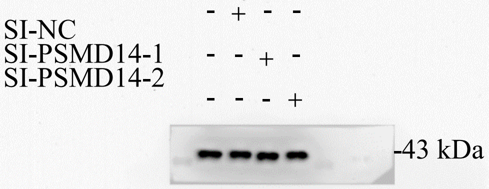

Figure 4A PSMD14

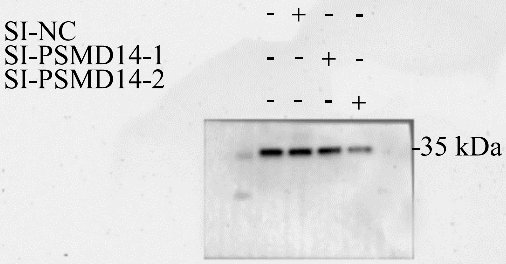

Figure 4A AKT1

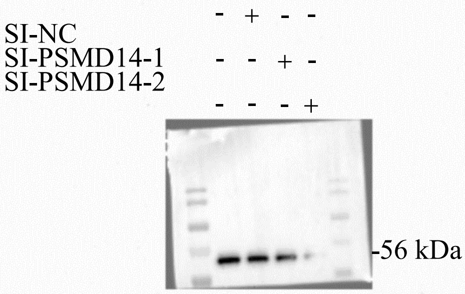

Figure 4A CDK4

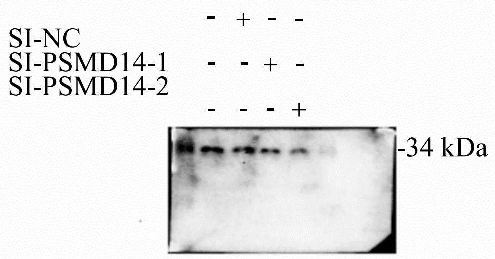

Figure 4C β-actin

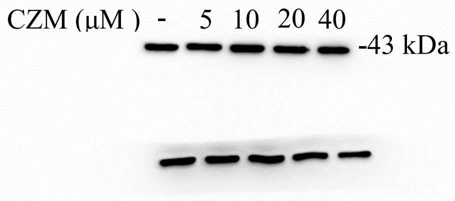

Figure 4C PSMD14

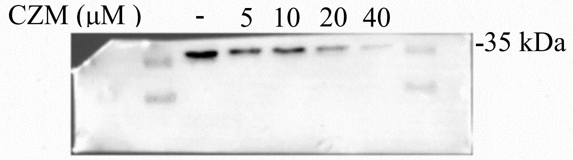

Figure 4C AKT1

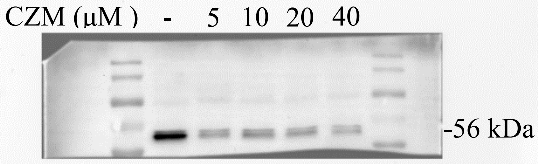

Figure 4C CDK4

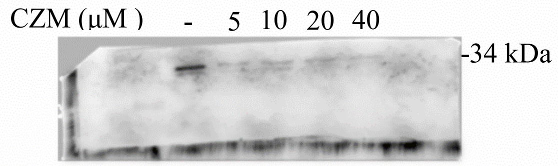

Figure 4E β-actin

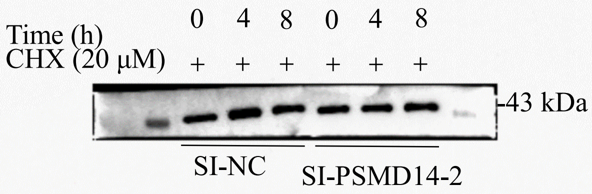

Figure 4E PSMD14

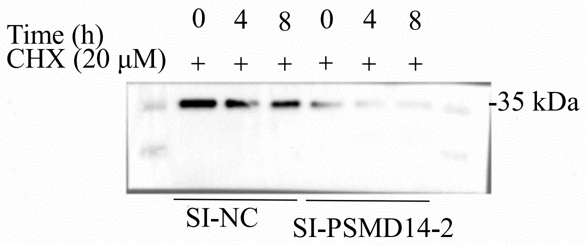

Figure 4E AKT1

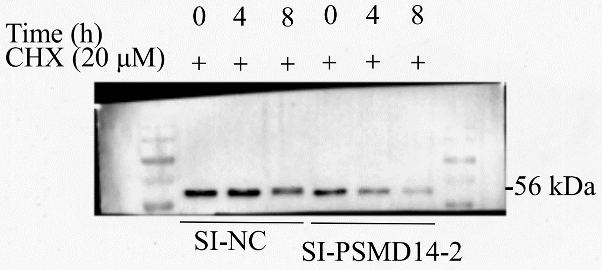

Figure 4E CDK4

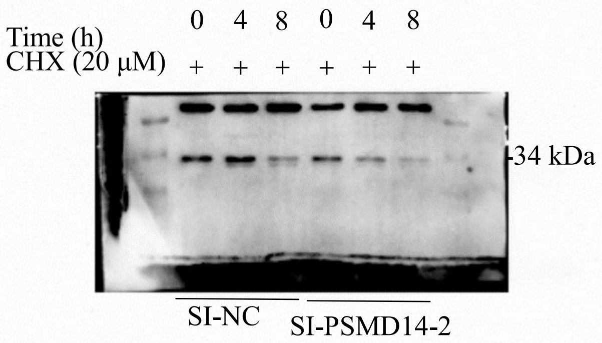

Figure 4G β-actin

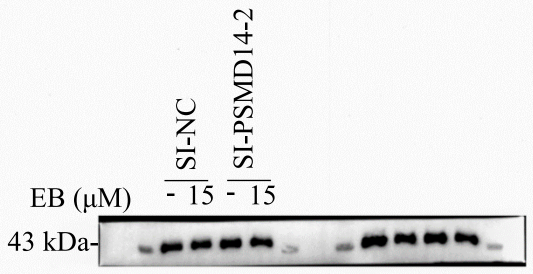

Figure 4G PSMD14

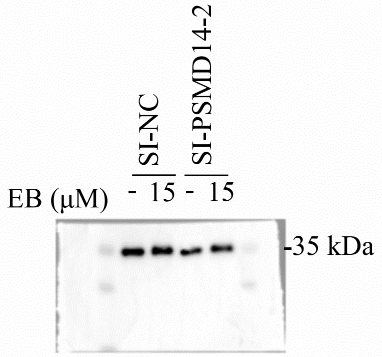

Figure 4G AKT1

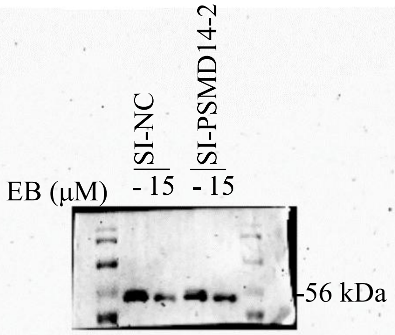

Figure 4G CDK4

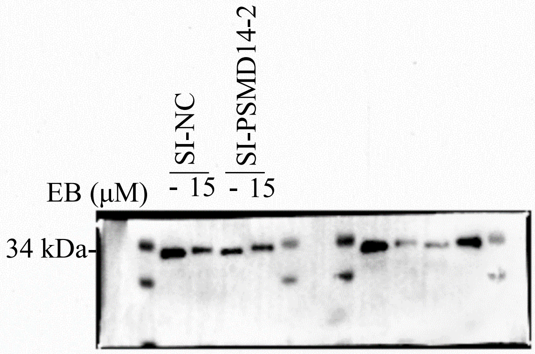

Supplement: CB-007-D5CB00197H-s001 [file CB-007-D5CB00197H-s001.pdf]
